# Emerging role of lncRNAs in osteoarthritis: An updated review

**DOI:** 10.3389/fimmu.2022.982773

**Published:** 2022-10-11

**Authors:** Rongliang Wang, Hoi Ting Shiu, Wayne Yuk Wai Lee

**Affiliations:** ^1^ Department of Orthopaedics and Traumatology, Faculty of Medicine, Prince of Wales Hospital, The Chinese University of Hong Kong, Hong Kong, China; ^2^ Li Ka Shing Institute of Health Sciences, The Chinese University of Hong Kong, Hong Kong, China; ^3^ SH Ho Scoliosis Research Laboratory, Joint Scoliosis Research Center of the Chinese University of Hong Kong and Nanjing University, The Chinese University of Hong Kong, Hong Kong, China

**Keywords:** long non-coding RNA, osteoarthritis, pathogenesis, biomarkers, therapeutic strategies

## Abstract

Osteoarthritis (OA) is a prevalent joint disease, which is associated with progressive articular cartilage loss, synovial inflammation, subchondral sclerosis and meniscus injury. The molecular mechanism underlying OA pathogenesis is multifactorial. Long non-coding RNAs (lncRNAs) are non-protein coding RNAs with length more than 200 nucleotides. They have various functions such as modulating transcription and protein activity, as well as forming endogenous small interfering RNAs (siRNAs) and microRNA (miRNA) sponges. Emerging evidence suggests that lncRNAs might be involved in the pathogenesis of OA which opens up a new avenue for the development of new biomarkers and therapeutic strategies. The purpose of this review is to summarize the current clinical and basic experiments related to lncRNAs and OA with a focus on the extensively studied H19, GAS5, MALAT1, XIST and HOTAIR. The potential translational value of these lncRNAs as therapeutic targets for OA is also discussed.

## Introduction

Osteoarthritis (OA) is a prevalent joint disease in aging and obese populations, resulting in joint pain, stiffness, and movement limitation (1). It has been estimated that OA affects more than 240 million people all over the world which is projected to double in the next 20 years (2). OA is regarded as one of the leading causes of major health and socioeconomic burdens in many countries (3). OA was once considered as a disease of articular cartilage alone. However, it is now generally believed that OA pathogenesis is associated with pathological changes of other joint tissues, such as synovial inflammation, subchondral bone remodeling and meniscal degeneration (4, 5). Risk factors, such as aging, obesity, trauma, genetic predisposition, and bone density, have been implicated in the onset and development of OA (6). Despite these well documented factors and other routinely used clinical parameters such as patient history and radiographic examination, there is still a lack of sensitive approach to detect OA at its reversible stage (7, 8). In the clinics, multiple conservative treatments are available at the early stage of OA, such as physical measures or pharmacological anti-inflammatory and analgesic drugs (6). Surgical interventions, such as osteotomies and total replacement surgeries are served as the ultimate therapeutic options to rehabilitate the persistent pain and functional limitations of patients suffering from severe OA (9, 10). Obviously, these approaches are not able to halt or the progressive degeneration in the joints. Collectively, a better understanding of the molecular mechanism underlying this complex pathogenesis will provide an insight into the development of more specific and sensitive biomarkers as well as disease-modifying drugs for OA prevention and treatment (11).

In human genome, approximately 2% of genome is made up of protein-coding genes. The remaining 98% genome was thought to be nonfunctional evolutionary leftovers. It is now evidenced that these widely distributed non-coding genomes can be classified into two groups, namely short (< 200 nucleotides) and lncRNAs (> 200 nucleotides) which have diverse biological functions in various human diseases (12). In general, lncRNAs modulate the expression of target genes or the activity of downstream pathways by direct binding to DNA, RNA and proteins (13). Increasing evidence reveals that there are differential expressions of lncRNAs at cellular and tissue levels in human OA condition (14), suggesting the undefined roles of lncRNAs in OA development and progression (15), and potentially a new class of biomarkers for OA (16).

To supplement our current understanding as summarized in previous reviews and to update the landscape of lncRNAs research in OA (17, 18), this review takes a more comprehensive approach to critically review the current findings about the role of lncRNAs in OA pathobiology and diagnosis with emphasis on those extensively studied lncRNAs, including lncRNA H19, GAS5, MALAT1, XIST and HOTAIR and their effects on various joint tissues, and to propose novel treatment strategies *via* targeting lncRNAs.

This review on clinical and basic studies was conducted to provide a current understanding about the lncRNAs research on multiple joint tissues of OA pathogenesis through searching published articles on the PubMed, Google Scholar, and ScienceDirect databases from February 2003 to August 2022. The searching keywords include (“long non-coding RNA” OR “lncRNA”) AND (“osteoarthritis” OR “arthritis” OR “osteoarthritis treatment”) AND (“plasma” OR “synovial fluid” OR “body fluid” OR “cartilage” OR “synovium” OR “subchondral bone” OR “meniscus” OR “chondrocyte” OR “synoviocyte” OR “osteoblast” OR “exosome” OR “nanoparticle” OR “siRNA” OR “Gene-editing”).

## Classification and function of lncRNAs

**Figure 1:**
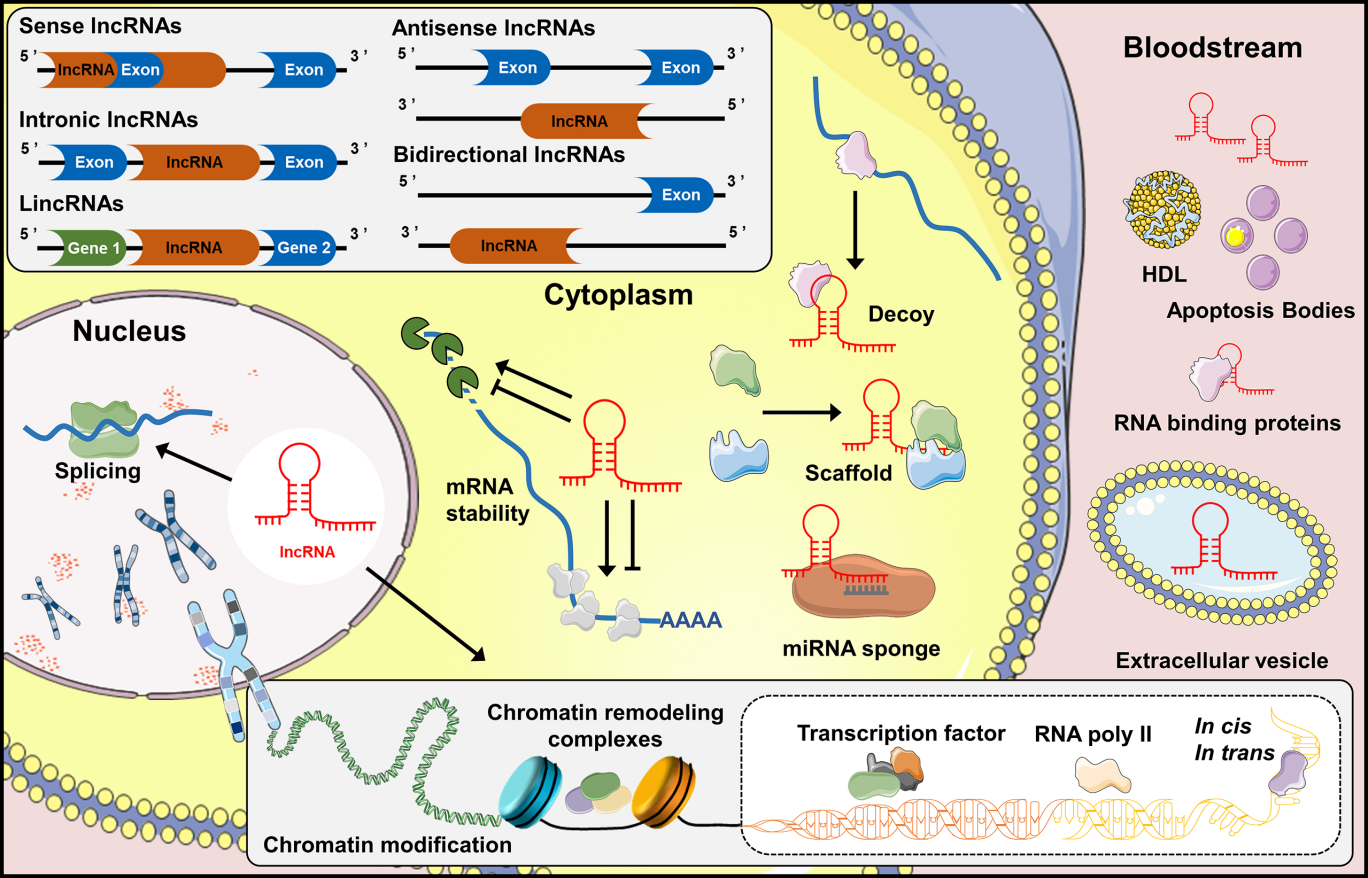
Biogenesis and function of lncRNAs. Classification of lncRNAs into five classes: sense lncRNAs, intronic lncRNAs, lincRNAs, antisense lncRNAs and bidirectional lncRNAs, based upon their genomic locations and transcription. LncRNAs regulate the expression of genes in the cytoplasm by interacting directly with microRNAs (miRNAs) or proteins, and stabilizing mRNA transcripts. Noncoding transcripts in the nucleus are known to regulate gene expression at the level of chromatin modification, transcription and post-transcriptional processing. In addition, lncRNAs are considered as biomarkers or participant in tissue crosstalk by entering the bloodstream directly, or bound to carrier proteins, even incorporated into extracellular vesicles which can be further released into bloodstream.

One common classification of lncRNAs is based on their positions to protein-coding genes: (i) Sense lncRNAs and (ii) antisense IncRNAs are those overlap with the same and opposite strand of coding genes, respectively; (iii) Intronic lncRNAs are those locate in the same intronic region of protein-coding genes. While (iv) bidirectional lncRNAs are transcribed from the same promoter as the protein-coding genes, but in the opposite direction and (v) long intergenic noncoding RNAs (lincRNAs) locate in the genomic interval between two genes (19) (**Figure 1**). In addition, lncRNAs can be further classified by their interactions with targets, including decoy lncRNAs, guide lncRNAs, scaffold lncRNAs, stabilizing lncRNAs and competitive endogenous-lncRNAs. Decoy lncRNAs sequester DNA-binding proteins to limit their bindings to DNA recognition elements. Guide lncRNAs recruit chromatin remodeling agents to impart specificity to genomic locations through either DNA-protein or RNA-DNA recognition. While scaffold lncRNAs join several proteins together in a complex, and stabilizing lncRNAs bind to target mRNA transcripts, stabilize and promote their translations. Competitive endogenous-lncRNAs (ceRNAs) or ‘RNA sponges’ compete with miRNAs to limit their effects on protein-coding mRNA targets (20).

Extensive research over the past decade has deciphered various biological functions of lncRNAs (21). In general, lncRNAs regulate gene expression *via* chromatin modification, transcription and post-transcriptional processes (22). During chromatin modification, lncRNAs recruit chromatin remodeling complexes to specific chromatin loci (23). Transcriptional regulation is the core role of lncRNAs in which they serve as pervasive enhancers or promoters of transcription. In addition, lncRNAs also behave as RNA binding proteins, transcription factors and RNA polymerase (RNAP) II in regulating the initiation of transcription (21). During post-transcriptional regulation, lncRNAs mediate mRNA dynamics in both cis- and trans-targets (24). Overall, lncRNAs serve as master regulators of gene expression, and it is not surprising that the value of lncRNAs in key aspects of OA progression has attracted considerable attention.

## Overview of lncRNAs in OA pathogenesis

Currently, most of the studies focused on the lncRNAs functions in OA cartilage/chondrocyte. Given that OA is a disease of the whole joint (25), it is of clinical value to provide an overview regarding the lncRNAs expression in different joint tissues. The section summarizes some recent key findings about the dysregulation of lncRNAs expression and their potential biological roles in cartilage degradation, synovial inflammation, dysfunction of subchondral bone homeostasis and meniscus injury. The full list of literature search can be referred to **Table 1**.

**Table 1:** The dysregulated lncRNA in clinical OA samples.

LncRNA	Human tissue/cells	Expression in OA	Potential targets	Cellular process	Proposed molecular mechanism	References
*H19*	OA Cartilage	Upregulated	COL2A1, COL9A1, COL10A1, CILP, and HTRA1	ECM anabolism	miR675 host	(26)
	OA chondrocyte	Upregulated	PCNA, CyclinD1, and cleaved Caspase 3	Cell proliferation	Interaction with miR106-5p	(27)
	OA Cartilage	Upregulated	IL-38	Inflammatory response	Interaction with p53	(28)
	OA Cartilage	Upregulated	Bax and Bcl2	Cell apoptosis	Interaction with miR140-5p	(29)
			COL2A1, MMP1, and MMP13	ECM degradation		
			ALP, OCN, and BSP	Ossification		
	OA synovium	Upregulated	–	–	–	(30)
*GAS5*	OA chondrocyte	Upregulated	MMP2, MMP3, MMP9, MMP13, and ADAMTS4	ECM degradation	Interaction with miR21 in autophagy	(31)
	OA chondrocyte	Upregulated	Bax and Bcl2	Cell apoptosis	Interaction with miR34a	(32)
	OA Cartilage	Upregulated	Caspase 3, Bax, and Bcl 2	Cell apoptosis	Interaction with miR137	(33)
	OA synovium	Downregulated	Caspase 3, and Bax	Cell apoptosis	–	(34)
*MALAT1*	OA chondrocyte	Upregulated	ADAMTS5, COL2A1, ACAN, and COMP	ECM degradation	Interaction with miR145	(35)
	OA Cartilage	Upregulated	OPN	Cell proliferation	Regulated PI3K/Akt pathway by interacting with miR127-5p	(36)
	OA synoviocytes	Upregulated	IL-6 and CXCL8	Inflammatory response	–	(37)
	OA Subchondral bone	Upregulated	PGE2	Inflammatory response	–	(38)
	OA Cartilage	Upregulated	Cleaved caspase3 and Bcl2	Cell apoptosis	Regulated DNMT3A by interacting with miR149-5p	(39)
			COL2 and aggrecan	ECM degradation		
	OA Cartilage	Upregulated	Cleaved caspase3 and Bcl2	Cell apoptosis	Regulated GNG5 by interacting with miR675-3p	(40)
			COL2A1 and MMP13	ECM degradation		
			IL-6 and IL-8	Inflammatory response		
	OA Cartilage	Upregulated	CXCR4	Cell proliferation	Regulated MAPK signaling by interacting with miR211	(41)
	OA synovium (Macrophagy)	Upregulated	IL‐4, IL-6, IL-10, IL‐1β, and TNF‐α	Inflammatory response	Interaction with miR376	(42)
			ICAM1, MMP3, MMP9, and MRP8	Migration		
			OPN, ACAN, and COL2 in chondrocyte	Crosstalk		
*HOTAIR*	OA Cartilage	Upregulated	Bcl2, cleave caspase3, p62 and LC3B	Cell apoptosis	Regulated ADAM10 by interacting with miR222-3p	(43)
			COL2, COL10, SOX9, and MMP13,	ECM degradation		
			IL-6, IL-10 and TNF-α	Inflammatory response		
	OA Cartilage	Upregulated	Cleaved caspase3, Survivin, Bcl2 and Bax	Cell apoptosis	Regulated BCL2L13 by interacting with miR130a-3p	(44)
	OA Cartilage	Upregulated	IL-1β and TNF-α	Inflammatory response	Regulated STGB by interacting with miR1277-5p	(45)
			Aggrecan and COL2	ECM degradation		
	OA Cartilage	Upregulated	Aggrecan, COL2, MMP13 and MMP9	ECM degradation	Regulated CXCL12 by interacting with miR107	(46)
			–	Cell apoptosis		
	OA chondrocyte	Upregulated	ADAMTS5 MMP13, ADAMTS5, COL2, and ACAN	ECM degradation	Interaction with promotor	(47)
					
	OA Cartilage	Upregulated	Cleaved caspase3, cleaved caspase9 and Bax	ECM degradation	Regulated FUT2/WNT aixs by interacting with miR17-5p	(48)
				Cell apoptosis	
	OA Cartilage	Upregulated	COL2, MMP9, MMP13, TIMP3, ACAN and ADAMTS5	ECM degradation	Regulated WIF1/WNT pathway	(49)
*HOTTIP*	OA chondrocyte	Upregulated	HoxA cluster	–	Epigenetic regulation	(50)
	OA Cartilage	Upregulated	–	Cell proliferation	Regulated FRK by interacting with miR 663a	(51)
*CIR*	OA Cartilage	Upregulated	MMP13, ADAMTS5, COL2, COL1, and ACAN	ECM degradation	Vimentin inhibition	(52)
	OA chondrocyte	Upregulated	MMP13	ECM degradation	Interaction with miR27	(53)
	OA Cartilage	Upregulated	COL2A1, and MMP13	ECM degradation	Activating autophagy	(54)
*MSR*	OA Cartilage	Upregulated	COL2A1, ACAN, MMP13, and ADAMTS5	ECM degradation	Regulated TMSB4 by interacting with miR152	(55)
*PVT1*	OA Cartilage	Upregulated	COL2, ACAN, MMP3, MMP9 and MMP13	ECM degradation	Interaction with miR149	(56)
			PGE2, NO, IL-6, IL-8, and TNF-α	Inflammatory response		
	OA Cartilage	Upregulated	Cleaved caspase3 and autophagy	Cell apoptosis	Regulated TRAF3 by interacting with miR27b-3p	(57)
	OA synovium	Upregulated	Caspase 3, and Bax	Cell apoptosis	–	(34)
*Nespas*	OA chondrocyte	Upregulated	COL2, COL1, MMP2 and MMP13	ECM degradation	Interaction with miRNAs	(58)
*UCA1*	OA Cartilage	Upregulated	COL2, COL4, and MMP13	ECM degradation	Interaction with miR204-5p	(59)
			–	Cell proliferation		
*DANCR*	OA Cartilage	Upregulated	Caspase3 and Bcl2	Cell apoptosis	Regulated SphK2 by competing with miR577	(60)
	OA Cartilage	Upregulated	IL-6 and IL-8	Inflammatory response	Regulated JAK2/STAT3 signaling by interacting with miR216a-5p	(61)
	OA Cartilage	Upregulated	IL-1, IL-6, IL-8, and TNF-α	Inflammatory response	Regulated DANCR by interacting with miR19a	(62)
			–	Cell apoptosis		
*LncHIFCAR*	OA Cartilage	Upregulated	MMP1, MMP3 and MMP13	ECM degradation	Regulated HIF-1α, HIF-1α target genes, and PI3K/AKT/mTOR pathway	(63)
			TNF-α and IL-6	Inflammatory response		
			Bcl2, Bax, and Cytochrome C	Cell proliferation		
*FAS-AS1*	OA Cartilage	Upregulated	COL2, MMP1 and MMP13	ECM degradation	–	(64)
			–	Cell proliferation	–	
*FOXD2-AS1*	OA Cartilage	Upregulated	CCND1	Cell proliferation	Interaction with miR206	(65)
*p21*	OA chondrocyte	Upregulated	Bcl2, and Bax	Cell apoptosis	Interaction with miR451	(66)
*TM1P3*	OA chondrocyte	Upregulated	MMP13	ECM degradation	Regulated TGF-β signaling by interacting with miR22	(67)
*TNFSF10*	OA chondrocyte	Upregulated	IL-6 and IL-8	Inflammatory response	Regulated FGFR1 by interacting with miR376-3p	(68)
			–	Cell proliferation		
			–	Cell apoptosis		
*LINC01534*	OA Cartilage	Upregulated	MMP3, MMP9, MMP13, COL2 and aggrecan	ECM degradation	Interaction with miR140-5p	(69)
			NO, PGE2, TNF-α, IL-6, and IL-8	Inflammatory response		
*NKILA*	OA Cartilage	Upregulated	Bcl2, Bax, and cleaved caspase3	Cell apoptosis	Regulated SP1/NF-κB axis by interacting with miR145	(70)
*LINC00461*	OA Cartilage	Upregulated	IL-6, IL-10	Inflammatory response	Interaction with miR30a-5p	(71)
			COL2, MMP2, MMP3 and MMP13	ECM degradation		
			–	Cell proliferation		
*LOXL1-AS1*	OA Cartilage	Upregulated	Cleaved Caspase 3, Cleaved Caspase 9, and Bax	Cell apoptosis	Regulated KDM5C by interacting with miR423-5p	(72)
			IL-6, IL-8	Inflammatory response		
*PCAT-1*	OA chondrocyte	Upregulated	Cleaved Caspase3, Bcl2, and Bax	Cell apoptosis	Interaction with miR27b-3p	(73)
*ARFRP1*	OA Cartilage	Upregulated	CCND1, Bcl2, and Bax	Cell apoptosis	Regulated TLR4/NF-κB axis by interacting with miR15a-5p	(74)
			TNF-α, IL-6, and IL-1β	Inflammatory response		
*TUG1*	OA Cartilage	Upregulated	MMP13, COL2 and aggrecan	ECM degradation	Regulated FUT1 by interacting with miR17-5p	(75)
			–	Cell apoptosis		
*LINC00671*	OA Cartilage	Upregulated	Col2A1, Aggrecan, MMP3, MMP13, ADAMTS4, and ADAMTS5	ECM degradation	Regulated ONECUT2/Smurf2/GSK-3β axis	(76)
*RMRP*	OA Cartilage	Upregulated	–	Cell proliferation	Regulated CDK9 by interacting with miR206	(77)
*KCNQ1OT1*	OA Cartilage	Upregulated	IL-1β, TNF-α and IL-6	Inflammatory response	Regulated TCF4 by interacting with miR211-5p	(78)
			COL2, COL10, and MMP13	ECM degradation		
	OA Cartilage	Downregulated	COL2, and MMP13	ECM degradation	Regulated TRPS1 by interacting with miR126-5p	(79)
			–	Cell proliferation		
*RP11-364P22.2*	OA Cartilage	Upregulated	Col2A1, Aggrecan, and MMP13	ECM degradation	Regulated ATF3	(80)
			Caspase3, and NF-κB	Cell apoptosis		
*Cox2*	OA Cartilage	Upregulated	Ki67 and PCNA	Cell proliferation	Regulated Wnt/β-catenin pathway by interacting with miR150	(81)
			Caspase3, Caspase9, and Bax	Cell apoptosis		
*CASC19*	OA Cartilage	Upregulated	IL-6, IL-8, and TNF-α	Inflammatory response	Regulated DDX6 by interacting with miR152-3p	(82)
			–	Cell apoptosis		
*MIR22HG*	OA Cartilage	Upregulated	COL2A1, ACAN, MMP13, ADAMTS5	ECM degradation	Interaction with miR9-3p	(83)
			–	Cell apoptosis		
*LINC01385*	OA Cartilage	Upregulated	IL-6, TNF-α, PGE_2_	Inflammatory response	Interaction with miR 140-3p/TLR4 axis	(84)
*LINC00707*	OA Cartilage	Upregulated	–	Cell apoptosis	Interaction with miR199-3p	(85)
	OA Cartilage	Upregulated	–	Cell apoptosis	Regulated FSHR by interacting with miR330-5p	(86)
			COL2, ACAN, MMP13, MMP3	ECM degradation		
			IL-6, TNF-α	Inflammatory response		
*LINC00680*	OA Cartilage	Upregulated	–	Cell proliferation	Regulated SIRT1 by interacting with IGF2BP2	(87)
			COL2, ACAN, MMP13,	ECM degradation		
*PILA*	OA Cartilage	Upregulated	MMP13, MMP3, ADAMTS4	ECM degradation	Regulated TAK1/NF-κB aixs by interacting with PRMT1	(88)
			–	Cell apoptosis		
*DLEU1*	OA Cartilage	Upregulated	COL2, ACAN, ADAMTS5 and MMP3	ECM degradation	Interaction with miR671-5p	(89)
			IL‐1, IL‐6, and TNF-α	Inflammatory response		
*MEG3*	OA Cartilage	Downregulated	VEGF	Angiogenesis	–	(90)
	OA chondrocyte	Downregulated	Ki67 and PCNA	Cell proliferation	Regulated FOXO1 by interacting with miR361-5p	(91)
			Bcl2 and Bax	Cell apoptosis		
			MMP13, ADAMTS5, COL2, ACAN	ECM degradation		
*CILinc01*	OA chondrocyte	Downregulated	IL-6	Inflammatory response	–	(92)
*CILinc02*	OA Cartilage	Upregulated	IL‐1, IL‐6, and IL‐17	Inflammatory response	–	(93)
			TIMP1, MMP1 and MMP13	ECM degradation	–	
			–	Cell apoptosis	–	
*UFC1*	OA Cartilage	Downregulated	–	Cell proliferation	Interaction with miR34a	(94)
*SNHG5*	OA Cartilage	Downregulated	SOX2	Cell proliferation	Interaction with miR26a	(95)
	OA Cartilage	Downregulated	MMP13, ADAMTS5, COL3 and ACAN	ECM degradation	Regulated autophagy by interacting with miR141-3p	(96)
			Cleaved caspase3	Cell apoptosis		
	OA Cartilage	Downregulated	Cleaved caspase3, and cleaved caspase9	Cell apoptosis	Regulated H3F3B by interacting with miR10a-5p	(97)
			COL2, and ADAMTS5	ECM degradation		
	OA Cartilage	Upregulated	MMP13 and ADAMTS5	ECM degradation
					Regulated TGFBR3 by interacting with miR181a‐5p	(98)
Caspase3	Cell apoptosis					
*HOTAIRM1-1*	OA Cartilage	Downregulated	–	Chondrogenic differentiation	Regulated BMPR2/MAPK aixs by interacting with miR125b	(99)
			Cleaved caspase3, cleaved caspase9, Bcl2 and Bax	Cell apoptosis		
			COL2, COL10, and aggrecan	ECM degradation		
*LINC00341*	OA Cartilage	Downregulated	Bcl2, and Bax	Cell apoptosis	Regulated YAF2 by interacting with miR141	(100)
*DNM3OS*	OA Cartilage	Downregulated	Cleaved caspase3, Bcl2, and Bax	Cell proliferation	Regulated IGF1 by interacting with miR126	(101)
*PART1*	OA Cartilage	Downregulated	Cleaved caspase3, cleaved caspase9 and Bax	Cell apoptosis	Regulated TGFBR2/Smad3 axis by interacting with miR590-3p	(102)
	OA Cartilage	Downregulated	MMP13, COL2, and ACAN	ECM degradation	Regulated SOX4 by interacting with miR373-3p	(103)
			Bcl2, Bax and cleaved caspase3	Cell apoptosis		
*NEAT1*	OA Cartilage	Downregulated	ACAN, Col2a1, MMP3, MMP13, and ADAMTS5	ECM degradation	Regulated SOX5 by interacting with miR373-3p	(104)
			IL-1, TNF-α, IL-6, and IL-8	Inflammatory response		
			–	Cell apoptosis		
	OA Cartilage	Upregulated	MMP3, MMP9, and MMP13	ECM degradation	Regulated PLA2G4A by interacting with miR543	(105)
			IL-6, and IL-8	Inflammatory response		
			p-Akt1 and Bcl2	Cell proliferation		
*LINC00662*	OA chondrocyte	Downregulated	TNF‐α, IL‐6, and IL‐8	Inflammatory response	Regulated GPR120 by interacting with miR15b-5p	(106)
			Cleaved caspase3, cleaved caspase9 and Bax	Cell apoptosis		
*OIP5-AS1*	OA Cartilage	Downregulated	IL-6, IL-8, and TNF-α	Inflammatory response	Regulated PGRN by interacting with miR29b-3p	(107)
			Bax	Cell apoptosis		
*LINC00623*	OA Cartilage	Downregulated	MMP13, and COL2	ECM degradation	Regulated HRAS/MAPK axis by interacting with miR101	(108)
			Cleaved caspase3, and cleaved caspase7	Cell apoptosis		
*SNHG7*	OA Cartilage	Downregulated	Cleaved Caspase3, Cleaved Caspase7	Cell apoptosis	Regulated SNHG7/PPARγ axis by interacting with miR214-5p	(109)
			IL-1β, TNF-α and IL-6	Inflammatory response		
*ROR*	OA Cartilage	Downregulated	COL2, ACAN, MMP13 and COL10	Chondrogenesis	Regulated SOX9 by interacting with miR138 and miR145	(110)
*OIP5-AS1*	OA Cartilage	Downregulated	Caspase 3, Caspase 9, Bax, and Bcl2	Cell apoptosis	Interaction with miR30a-5p	(111)
			IL-6, IL-8, and TNF-a	Inflammatory response		
*FGD5-AS1*	OA Cartilage	Downregulated	–	Cell apoptosis	Regulated TGFBR2 by interacting with miR302d-3p	(112)
*MCM3AP-AS1*	OA Cartilage	Downregulated	–	Cell apoptosis	Regulated SIRT1 by interacting with miR138-5p	(113)
*MEG8*	OA Cartilage	Downregulated	Caspase3	Cell apoptosis	Regulated PI3K/AKT signaling	(114)
			IL-6 and TNF-α	Inflammatory response		
*ZFAS1*	OA Cartilage	Downregulated	ROS, SOD, and Catalase	Oxidative stress	Regulated NRF2 by interacting with miR1323	(115)
			IL-1β, TNF-α and IL-6	Inflammatory response		
			–	Cell apoptosis		
*GACAT3*	OA synoviocytes	Upregulated	Caspase3	Cell proliferation	Regulated STAT3	(116)
*ANRIL*	OA synoviocytes	Upregulated	Cleaved caspase3, Bax, and Bcl2	Cell proliferation	Regulated DUSP4 by interacting with miR122-5p	(117)
*PCGEM1*	OA synoviocytes	Upregulated	PARP and caspase9	Cell proliferation	Interaction with miR770	(118)
	OA synoviocytes	Upregulated	Chondrocyte apoptosis and cartilage matrix degradation	Crosstalk	Interaction with miR142-5p	(119)
*AK094629*	OA synovium	Upregulated	IL-6	Inflammatory response	Regulated MAP3K4	(120)
*IGHCγ1*	PBMCs	Upregulated	IL-6 and TNF-α	Inflammatory response	Regulated TLR4/NF-κB axis by interacting with miR6891-3p	(121)
*AC005165.1*	OA Subchondral bone	Downregulated	–	–	Regulated FRZB/WNT signaling	(122)
*LOC107986251*	OA Menisci	Upregulated	–	–	Regulated SESN3 by interacting with miR212-5p	(123)

COL, Collagen; CILP, Cartilage intermediate layer protein; ECM, Extracellular matrix; PCNA, Proliferating cell nuclear antigen; MMPs, Matrix metalloproteinases; ALP, Alkaline phosphatase; OCN, Osteocalcin; BSP, Bone sialoprotein; ACAN, Aggrecan; ADAMTS, A disintegrin and metalloproteinase with thrombospondin motifs; COMP, Cartilage oligomeric matrix protein; PGE2, Prostaglandin E2; OPN, Osteopontin; TIMPs, Tissue inhibitor of metalloproteinases; CXCL, C-X-C Motif Chemokine Ligand; CXCR, C-X-C chemokine receptor; MRP, Multidrug resistance-associated protein; CCND1, Cyclin D1; VEGF, Vascular endothelial growth factor; ROS, Reactive oxygen species; SOD, Superoxide Dismutase; PBMCs, Peripheral Blood Mononuclear Cells.

### lncRNAs in Cartilage

Cartilage is an integral part of the skeletal system and is mostly composed of chondrocytes. Chondrocytes can secrete cartilage matrix and maintain joint activity (124), making this cell type indispensable to the dynamic and continuous processes of extracellular matrix (ECM) deposition and remodeling to maintain homeostasis of cartilage (125). However, such balance is disrupted in OA, and finally resulting in degeneration of cartilage matrix (notably type II collagen, COL2), production of fibrous ECM, aberrant proliferation, senescence and hypertrophy of chondrocytes, as well as secretion of inflammatory cytokines (126). Previous studies described the abnormal expression of lncRNAs in OA cartilages or chondrocytes, indicating the probable link between lncRNAs and the aberrant chondrocyte function (127, 128). Liu and colleagues are one of the pioneer groups to profile lncRNA in human OA cartilage tissues, providing a new insight into the mechanism of cartilage injury and the progression of ECM degradation (52). Similarly, Hoolwerff and colleagues reported the differential expression of lncRNAs with OA pathophysiology in cartilage, and they discussed the potential of antisense lncRNA P3H2-AS1 on collagen chain assembly in lesioned OA cartilage *via* the regulation of P3H2 expression (129). On the other hand, Pearson et al. identified 125 lncRNAs were differentially expressed upon IL-1β stimulation in primary human OA chondrocytes. Amongst, two novel lncRNAs, namely ClLinc01 and ClLinc02, were found to mediate the secretion of proinflammatory cytokines in IL-1 stimulated human chondrocytes, suggesting that some lncRNAs might mediate the response of chondrocytes to inflammation and inflammation-driven cartilage degeneration within the OA joint (92). Of note, different types of cellular model, such as cartilage derived primary cell culture or immortalized cell line with or without prior stimulation, were used in previous studies to delineate the effects of various lncRNAs on chondrocytes (130). Whether these effects are associated with or even causative factors in OA development or progression requires further investigation with appropriate transgenic animal models.

### lncRNAs in Synovium

Synovium is a specialized connective membrane lining the inner surface of synovial joint capsules, and almost 75% of cells in the synovium are fibroblast-like synoviocytes (FLS) (131). Increasing evidence shows that FLS secretes proinflammatory cytokines which mediate the degradation of cartilage during OA progression (132), which has been speculated to be associated with disease progression (133). Till now, the effects of lncRNAs on OA synovium remains elusive. Early work by Xiang and colleagues identified the differential expressions of 17 lncRNAs in OA synovium of aged patients undergoing total knee replacement surgery, in which some of these lncRNAs were found to be related to immune response. The recruitment of younger control subjects requiring arthroscopic meniscectomy in this case-control study is ethically sound but not ideal to exclude the influences of the acute injury of meniscus on the lncRNAs in the synovial microenvironment (134). Li and colleagues focused on a hepatocellular carcinoma associated lncRNA (ANRIL) and found a higher level of ANRIL in the OA cartilage tissue when compared with that of normal cartilage tissue obtained from subjects requiring traumatic emergency amputation without OA or rheumatic arthritis. Then primary chondrocytes isolated from the collected cartilage tissues, and commercially available normal and OA synoviocytes were used to show differentially upregulated ANRIL expression in OA synoviocytes but not in OA chondrocytes. It appears that ANRIL dysregulation in OA is cell-type specific, affecting the proliferation of synoviocytes *via* binding to miR-122-5p (117). However, it should be noted that the information of the subjects where those chondrocytes and synoviocytes derived from (such as age and sex) were not provided, which should be taken into consideration.

### lncRNAs in Subchondral bone

Impaired mineralization is a pathological feature of osteoarthritic subchondral bone. Such distinct microstructural alterations, including sclerotic changes and osteophyte formation, are both believed to arise from elevated bone turnover with an increase in osteoblastic over osteoclastic activities (135). In addition, the subchondral bone is also considered as a major site of OA pain, likely due to the innervation with sensory neurons and vascular channels (136). From bone remodeling perspective, it is evidenced that several lncRNAs could regulate osteoblast and osteoclast activities, and there are attempts to modulate lncRNAs expression *in vivo via* various strategies (137). Therefore, it is of interest to ask whether aberrant subchondral bone remodeling in OA is associated with lncRNAs dysregulation. By comparing subchondral bone samples collected from hip and knee, Tuerlings and colleagues identified 21 lncRNAs differentially expressed between preserved and lesioned OA subchondral bone significantly. It is interesting to note that a further stratified analysis identified 15 lncRNAs were differentially expressed in knee samples but none in hip samples (122). These findings prompt to further research questions. 1) Whether lncRNAs differential expression in OA subchondral bone is site-specific and associated with aberrant mechanical loading? 2) What are the biological functions of these lncRNAs in OA subchondral bone remodeling? Further investigation on the effects of lncRNAs on osteoblasts, osteoclasts and osteocytes functions related to subchondral bone mineralization and remodeling is warranted to develop a more comprehensive understanding of the lncRNAs and their roles and therapeutic values in OA.

### lncRNAs in Meniscus

Meniscus is a crucial tissue for supporting the structure, stability, and biomechanical function of the knee joint (138). During OA progression, it undergoes various histopathological changes, including tears, calcification, and atypical cell arrangement (139). Till now, there is limited studies exploring the mechanism of meniscal pathogenesis in OA, and only two studies were found to investigate the expression level of lncRNAs in OA meniscus tissues. The work by Brophy and colleagues depicted the transcriptome profile in the meniscus between end stage OA patients and patients undergoing arthroscopic partial meniscectomy with no evidence of OA. The subjects in the OA group were older and had higher BMI. Twenty-six and 10 lncRNAs were found up- and down-regulated in the OA group, respectively. Lnc-RPL19-1 and lnc-ICOSLG-5 were highlighted because of their correlations with some cartilage disease related genes. qPCR was performed to validate the microarray results (140). Recently, Jiang and colleagues performed a whole-transcriptome profile of lncRNAs dysregulation using isolated meniscus cells from OA patients with and without IL-1β, suggesting a potential crosslink between menisci and cartilage during OA. Of note, LCN2 and RAB27B were consistently upregulated in both OA meniscus and IL-1β treated primary meniscus cells derived from three OA meniscus samples, and appears to be associated with OA severity (123). Although different samples were used in the analysis, these two works both illustrated the potential link between inflammatory phenotype in meniscus and lncRNAs, which is subjected to further investigation to confirm the molecular mechanisms and biological functions of these lncRNAs in OA meniscus injury.

## Clinical biomarkers of lncRNAs for OA diagnosis

In general, the secretion and transport of lncRNAs into extracellular environment are mediated by three manners (1): Direct release of extracellular RNAs by joint tissues and cells (2). Encapsulated in high density lipoprotein (HDL) or apoptosis bodies or associated with protein complexes (3). Packed in membrane vesicles, such as exosomes and micro-vesicles (141). In clinical research, serum and synovial fluid are often the preferred biological fluid samples for OA biomarker discovery (142). Recent detections of the extracellular lncRNAs in these biological fluids of OA subjects implicate that they might serve as alternative indicators for OA onset and progression (**Table 2**).

**Table 2:** LncRNAs as biomarkers for OA diagnosis.

LncRNA	Human Samples	Expression in OA	Sample size(Health vs OA)	AUC	Correlation	References
*ATB*	Serum	Downregulated	76 vs 98	0.8902	No significant association with the clinical data	(143)
*H19*	Peripheral Blood	Upregulated	100 vs 103	0.891	K-L grading, and Bone metabolism indexes	(144)
*DILC*	Plasma	Downregulated	52 vs 87	0.9321	IL-6	(145)
	Synovial Fluid	Downregulated	14 vs 22	–	–	
*FER1L4*	Plasma	Downregulated	49 vs 81	0.9221	IL-6	(146)
	Synovial Fluid	Downregulated	16 vs 19	–		
*ANCR*	Plasma	Downregulated	62 vs 46	0.8845	TGF‐β1	(147)
*MIR4435-2HG*	Plasma	Downregulated	58 vs 78	–	–	(148)
	Synovial Fluid	Downregulated		0.96		
*LUADT1*	Synovial Fluid	Downregulated	60 vs 60	–	–	(149)
*CAIF*	Synovial Fluid	Downregulated	60 vs 60	0.89	miR1246 and IL-6	(150)
*PMS2L2*	Synovial Fluid	Downregulated	62 vs 62	–	OA stages	(151)
*HOTAIR*	Synovial Fluid	Upregulated	13 vs 21	–	–	(152)
*CASC2*	Synovial Fluid	Upregulated	60 vs 60	–	miR93-5p	(153)
*CTBP1-AS2*	Synovial Fluid	Upregulated	62 vs 62	–	miR130a	(154)
*GAS5*	Synovial Fluid	Downregulated	45 vs 45	–	–	(155)
	Synovial Fluid	Downregulated	62 vs 62	–	–	(34)
	Peripheral Blood Mononuclear Cells	Downregulated	60 vs 67	–	–	(156)
*LINC00167*	Peripheral Blood Leukocytes	Downregulated	60 vs 60	0.879	No significant association with the clinical data	(157)
*PVT1*	Serum/Serum Exosomes	Upregulated	30 vs 30	–	miR93-5p	(158)
	Synovial Fluid	Upregulated	62 vs 62	–	–	(34)
*PCGEM1*	Synovial Fluid Exosomes	Upregulated	20 vs 42	0.879	OA Stages, and WOMAC Index	(159)

### Circulation

Previous studies have shown that there is a relationship between the blood level of lncRNAs and OA progression (**Table 2**). For instance, lncRNA DILC (145), and lncRNA FER1L4 (146) were also found to be closely associated with OA inflammatory condition in plasma. As ANCR is known to regulate TGF-β signaling, Li and colleagues proposed that the plasma levels of TGF-β1 and ANCR could differentiate OA patients from healthy control subjects. They found a higher TGF-β1 and a lower ANCR level in OA plasma (N=62) when compared with that of healthy controls (N=46), which was inversely correlated. The mean area under curve (AUC) for OA plasma TGF-β1 and ANCR were 0.8929 and 0.8845, respectively (147). However, it is not shown if combination of plasma TGF-β1 and ANCR could enhance the sensitivity and specificity. Zhou et al. indicated that the expression of lncRNA H19 was negatively correlated with bone metabolic index of OA patients, such as Procollagen I N-Terminal Propeptide (PINP), N-MID-Osteocalcin, bone Gla protein (BGP), and bone alkaline phosphatase (BALP). Particularly, lncRNA H19 is highly correlated with K-L grading, VAS, WOMAC and Lysholm scores, suggesting H19 was associated with disease severity in OA patients (144). These two biomarkers discovery studies show encouraging AUC value, however, discussion on confounding factors and validation with separate cohort were missing.

### Synovial fluid

Based on current findings, it is reasonable to speculate that the expression of lncRNAs is cell and tissue specific in OA joint. Therefore, the information from research on synovial fluid is likely to provide additional clues on the clinical values of lncRNAs as OA biomarkers. Qi and colleagues showed lower levels of CAIF in the synovial fluid collected from the hip and knee of OA patients, and CAIF was inversely and significantly correlated with IL-6 expression level (150). Meanwhile, Xiao and colleagues reported lower levels of lncRNA MIR4436-2HG in both plasma and synovial fluid of OA patients. The mean AUC for CAIF and MIR-4435-2HG were found to be 0.89 and 0.96, respectively. It is interesting to note that 1 or 3 months treatment including exercise, prescription of non-steroidal anti-inflammatory drugs (NSAIDs) and joint burden reduction seems to increase the plasma level of MIR-4435-2HG (148). Although the study design, the details of these treatment and the compliance were not mentioned, this preliminary result suggests that lncRNAs level in circulation could be modulated. In these studies, healthy volunteers were recruited as control group for the collection of synovial fluid. If the collections of synovial fluid from mild to moderate stages are also ethically feasible, it will be of clinical interest to determine the correlations between lncRNAs level in synovial fluid and OA severity and progression in order to explore the prognostic value of those selected lncRNAs.

### Others

LncRNAs in cells/extracellular carriers within the blood and synovial fluid are another sources of biomarker candidates (160). The expression profile of lncRNAs in peripheral blood leukocytes of OA patients showed that LINC00167 may serve as a potential early diagnosis marker for OA in clinical practice (157). In addition, lncRNA GAS5 in the peripheral blood mononuclear cells isolated from the knee of OA patients was also lower than that of healthy subjects, indicating a novel marker for occurrence and progression of OA (156). The first study of IncRNA profiles in human OA synovial exosomes by Wu et al. found that exosomal lncRNA PCGEM1 is a potential indicator to distinguish the early stage of OA from the late-stage. Moreover, the expression of lncRNA PCGEM1 in synovial exosome rather than that in plasma was found to be closely associated with the WOMAC Index (159).

## Biological functions of lncRNAs in OA pathogenesis

### lncRNA H19

H19 lncRNA is located on chromosome 11p15.5, and its transcription product, H19 RNA, primarily resides in cytoplasm (161). It is the first reported mammalian lncRNA (162), which is highly expressed during fetal stage but markedly down-regulated after birth. H19 was found to be upregulated in OA cartilage, and appears to be associated with the disease progression (26, 163, 164). In primary human chondrocytes, H19 and H19-derived miR675 increased the matrix production of differentiated chondrocytes *via* activating *COL2* transcription (165). Furthermore, H19 could regulate the proliferation and apoptosis of chondrocytes treated by IL-1b *via* sponging miR106a-5p (27). Meanwhile, lncRNA H19 upregulated IL-38, which is bound to IL- 36R and brought about suppression of knee joint inflammation in mouse chondrocytes (28). Inconsistent outcomes were observed in different *in vitro* models and upon different stimulations. Knockdown of lncRNA H19 could alleviate apoptosis and inflammatory response *via* sponging miR130a in LPS-stimulated human C28/I2 chondrocytea (166). Furthermore, the effect of H19 silencing suppressed the expression of matrix metalloproteinases (MMPs) family (MMP1 and MMP3) via targeting miR-140-5p in human HC-A chondrocyte cells , suggesting a protective role of H19 on the degradation of the chondrocyte extracellular matrix (29). Besides OA chondrocyte, H19 RNA level in OA synovial tissue was also found to be significantly higher those that in synovium of normal and trauma joint (30). However, there is a lack of strong evidence supporting that H19 RNA upregulation is a sign of inflammation of synovial FLSs nor polarization of synovial macrophages (167). Notably, rats FLS-derived exosomal lncRNA H19 was found to promote chondrocyte viability and migration, as well as inhibit ECM degradation in IL-1β-induced chondrocytes by targeting miR106b-5p expression (168). Altogether, these studies suggest that lncRNA H19 may play an essential role in the crosstalk between synovium and cartilage during OA progression, and H19-targeted therapy is expected to open new perspectives for OA management.

### lncRNA GAS5

The growth arrest-specific 5 (GAS5) lncRNA is located on chromosome 1q25.1 and consists of 12 exons with a short open reading frame (ORF) (169). Its name reflects its nature and predominant expression in growth-arrested cells (170). As such, GAS5 is mainly responsible for suppressing multiple anti-apoptotic genes, thereby enhancing the vulnerability of cells to pro-apoptotic signals (171). In OA cartilage, GAS5 was found to be upregulated with positive correlation pattern to the disease stages (172, 173). Overexpression of GAS5 was reported to increase the activity level of chondrocyte catabolism (several MMPs), and apoptosis (31). Meanwhile, GAS5 can serve as negative regulators for miR21 (31), miR34a (32), miR137 (33), miR144 (173) and miR27a (174). It is also evidenced that GAS5 could directly target KLF2 to alleviate LPS-induced inflammatory damage in murine chondrocytic ATDC5 cell line (175). On the contrary, the expression levels of GAS5 in synovial fluid and tissues were significantly lower in OA (34, 155), which possibly implicate different functions of GAS5 in OA synovium. Considering the small sample size (N=45) and a lack of *in vivo* functional analysis, future study is required to evaluate the function of GAS5 in OA synovium by including a clinical study with a larger sample size and experiments with appropriate animal models.

### lncRNA MALAT1

Metastasis-associated lung adenocarcinoma transcript 1 (MALAT1), also known as NEAT2 for nuclear-enriched abundant transcript 2, is transcribed by RNA polymerase II at human chromosome 11q13 (176). It is a highly abundant nuclear transcript localized to the nuclear speckles and have a longer half-life (9–12 h) than other lncRNAs owing to bipartite triple helix structure (177, 178). MALAT1 is upregulated in human OA cartilage and IL-1β-induced chondrocyte cells (35). Overexpression of MALAT1 in human chondrocytes inhibited cells viability and promoted cartilage ECM degradation through targeting miR145 (35). Also, lncRNA MALAT1 overexpression in human C28/I2 chondrocyte cells was proved to promote chondrocyte migration, inflammation suppression, and ECM degradation (179). Besides, MALAT1 could act as sponges for other miRNAs, like miR127-5p (36), miR150-5p (180) and miR146a (181), thus likely to play some regulatory roles in OA cartilage. It should be noted that lower level of MALAT1 was also reported in IL-1b stimulated rat chondrocytes, which enhanced cell proliferation and type II collagen (Col II) expression by blocking JNK signaling activation (182). In synovium, the synovial fibroblasts isolated from OA patients had a higher expression of MALAT1 compared with that of normal subjects, which could be owing to proinflammatory challenge in synoviocytes especially to IL-6 and CXCL-8 (37). It is worth mentioning that MALAT1 is the first lncRNA to be investigated in OA subchondral bone. Higher expression level of MALAT1 was reported in both knee and hip subchondral bone of patients with OA, and its expression in the osteoblasts appears to be associated with the production of inflammatory prostacyclins. Since the subchondral bone is considered to be an important site of OA pain, MALAT1 may play an important role in the development of OA bone pain and inflammation (38). Based on current evidence, it appears that MALTA1 plays more pro-inflammatory role in OA synovial and subchondral bone, which represents a potential candidate for research on OA pathogenesis and therapeutic target.

### lncRNA XIST

X-inactive specific transcript (XIST) encodes a 17-kb lncRNA which, despite being capped, spliced and polyadenylated, it is retained in the nucleus (183). lncRNA XIST and its associated chromatin modifying complex play vital roles in the regulation of the X-chromosome inactivation process (184). Emerging evidence indicates that it is correlated with the modification of ECM component of OA (185). XIST was upregulated in OA cartilage and promoted MMP-13 and ADAMTS-5 expression in human chondrocytes, indicating its role in ECM degradation through functioning as a ceRNA of miR1277-5p (186). Notably, the consistency results could be seen in the studies of XIST in terms of repressing the development of OA as indicated by different models. For instance, in IL-1b induced human C28/I2 chondrocyte cells, the knockdown of XIST expression suppressed the production of IL-6, TNF-α, PGE2 and NO through the interaction with miR130a (187). XIST regulated IL-1β-induced chondrocyte growth, apoptosis and ECM synthesis through sponging with miR-142-5p in human chondrosarcoma cell line SW1353 (188). Moreover, the silencing of XIST could promote cell viability but inhibit cell apoptosis through acting as a sponge for miR149-5p in human CHON-001 chondrocyte cell line (39). In addition, XIST expression was significantly upregulated in the OA synovium compared with that in normal synovium. More importantly, XIST/miR376c‐5p/OPN axis has been proven to modulate the inflammatory microenvironment in OA synovial macrophage, subsequently affecting chondrocyte apoptosis and ECM degradation (42).

### lncRNA HOTAIR

HOX transcript antisense RNA (HOTAIR) resides within the intergenic region in HOXC cluster on chromosome 12, and acts as a crucial modulator of chromatin re-modeling and transcriptional silencing (189). As an epigenetic agent, HOTAIR can interact with various factors, leading to genomic stability, proliferation, survival, invasion, migration, metastasis, and drug resistance (190). In OA cartilage, HORAIR was upregulated than that of normal samples (164). HOTAIR was reported as a promising promoter for ADAMTS-5 expression and ECM degradation in human OA articular chondrocytes (47). HOTAIR silencing reduced cartilage tissue damage in OA mice, and promoted the expression of collagen II and aggrecan in cartilage tissue, while inhibited the expression of MMP-13 and ADAMTS-5 by targeting miR-20b/PTEN axis in mouse primary chondrocytes (191). Interestingly, cumulative evidence shows that Wnt/β-catenin pathway might play a certain role in the pathogenesis of cartilage damage, and lncRNA HOTAIR could directly bind to miR17-5p and indirectly regulate FUT2/β-catenin axis in connection with OA progression, such as ECM degradation and cell apoptosis (48). Wnt inhibitory factor 1 (WIF-1), a key inhibitor of the Wnt/β-catenin pathway, could be directly modulated by HOTAIR and interfered with the activation of downstream pathway and relative genes expression on cartilage degradation in human chondrosarcoma cell line SW1353 (49). Overexpression of HOTAIR in human CHON-001 chondrocyte cell line could aggravate LPS-induced cell apoptosis and inflammatory cytokines influx, including IL-1β, IL-6, IL-8 and TNF-α. While blocking HOTAIR could suppress cleavage of caspase-3 and p62 proteins and elevated secretion of IL-6 and TNF-α *via* suppression of miR222-3p (43). Meanwhile, HOTAIR inhibited chondrocytes proliferation *via* sponging with other miRNAs, including miR130a-3p (44), miR1277-5p (45), miR107 (46), and miR221 (192). Therefore, all HOTAIR-related factors form a comprehensive regulatory network, suggesting the central role of HOTAIR in the physiology of chondrocytes during OA (130).

Collectively, the identification of disease-specific lncRNAs for OA pathophysiology, including H19, GAS5, MALAT1, XIST, HOTAIR and future identified lncRNAs, emphasized the general consistency of lncRNAs functions in various tissues, which might be further developed as lncRNAs-targeted therapies for OA treatment in the future.

## Targeting lncRNAs: A novel treatment strategy for OA?

**Figure 2:**
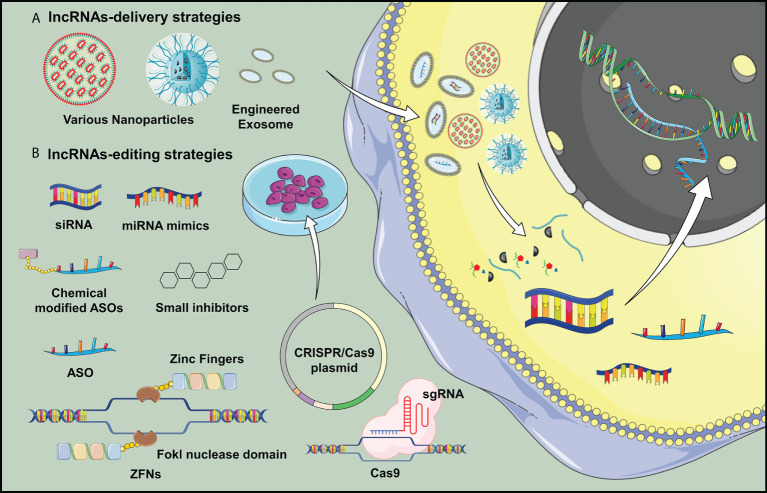
Potential delivery strategies for lncRNA H19 is proposed in OA treatment. **(A)** Nanotechnology and lncRNA-loaded exosomes could overcome the low efficiency of *in vivo* transgene lncRNA transfection, which would be applicable for widespread clinical application of gene therapy targeting lncRNAs. **(B)** Various transgene technologies may benefit lncRNA overexpression or downregulations *in vivo* studies, which opened a new door in studying the delivery of genetic material for OA treatment.

Based on current evidence, it is worthwhile to explore if targeting lncRNAs could be a novel strategy for preventing and/or treating OA. Till now, according to clinical trials registries (clincialtrials.gov), there is only one registered clinical trial studying the role of lncRNAs as biomarkers for OA articular microenvironment. Without relevant clinical studies can be included for discussion, we attempted to propose strategies developed for lncRNA delivery and targeting with reference to published animal studies (**Figure 2**).

Extracellular vesicles hold some promise to be a vehicle for selective delivery of target genes into tissues of interest (193). In animal study, intra-articular injection of exosomes with overexpressed lncRNA H19 is found to promote cartilage repair and restore OA joint homeostasis (194). Liu and colleagues highlighted the possible mechanism for OA therapy by cellular delivery of exosomal lncRNA KLF3-AS1, which could facilitate cartilage repair by promoting chondrocyte proliferation and migration and inhibiting apoptosis (195). Zhang and colleagues also reported that targeting lncRNA NEAT1 through artificial exosomes could be one of the options to elevate chondrocyte proliferation for OA treatment (196). Pan and colleagues confirmed the effect of MALAT1 on chondrocytes, which exhibited a slight cartilage damage and a smooth surface after intra-articular injection of LAMAT1 extracellular vesicles in OA animal model (179). In addition, the use of nanoparticles as an effective delivery vehicle for targeting lncRNAs provides a new therapeutic strategy owing to improved stability, biocompatibility, and high-dose therapeutic payloads (197). Recent advancement in lipid nanoparticles, polymeric nanocarrier and metal-based delivery system provides novel approaches for delivering of nucleic acids and lncRNAs-based therapeutic agents (198–200). At the time of writing, although nanoparticle delivery strategies for targeting lncRNAs in OA field has not been reported, therapeutic carriers, exosomes and nanomaterials pose enormous potential as vehicles loading gene-editing systems for OA treatment.

Considering upregulation of lncRNAs in OA pathogenesis appears to be the most common aberrant change, it is reasonable to propose approaches which can inhibit their expression or activity. Short interfering RNAs (siRNAs) is currently one of the *in vivo* feasible methods that has been shown to alleviate joint inflammation and decrease the expression of pro-inflammatory mediators by targeting lncRNA PVT1 in OA mice (201). Other *in vivo* approaches to regulate lncRNAs expression, such as locked nucleic acids (LNA) and ASOs have been shown to be effective to inhibit cancer progression (202, 203), which is pending for testing in OA animal models. Gene-editing enzymatic systems, such as zinc finger nucleases (ZFNs) and clustered regularly interspaced short palindromic repeats (CRISPR), are known far superior to RNAi technique for lncRNAs knockdown (204). Recently, some small molecule inhibitors are identified to systematically target lncRNA expression by masking the binding sites or disrupting the RNA structure (205).

## Conclusions and future direction

Increasing evidence indicates that lncRNAs are playing certain important roles associated with the pathological changes of OA joints through diverse actions on various joint components, which is exemplified by lncRNAs H19, GAS5, MALAT1, XIST and HOTAIR in this review.

The roles of lncRNAs have been mainly investigated with OA cartilage tissues and chondrocytes, and found to participate in the regulation of cartilage metabolism and chondrocyte function as a miRNA sponge regulating target genes expression. However, this kind of action and post-transcriptional regulation on target genes/proteins might not represent the whole picture of lncRNAs function in the context of OA. In addition, it should be admitted that the diverse methods employed in previous studies for lncRNA expression and functional analyses, such as the source of the testing cells, experimental procedures and even stimulation approaches, might lead to inconsistent findings.

In addition, the following questions remain elusive (1): the cause of lncRNAs dysregulation in the onset, development and progression of OA is still unclear. Whether the inflammation, hypoxia (26) or mechanical stress (206) are the major upstream factors leading to the aberrant expression of lncRNAs (2). Numerous miRNAs or proteins are reported to be downstream targets of lncRNAs, but their roles in line with lncRNAs dysfunction in OA pathogenesis remains largely unclear (3). In view of the diverse biological functions of lncRNAs, it is uncertain whether the effect of lncRNAs on the development and progression of OA is tissue- and/or cell-specific.

In view of the association with OA phenotypes, the clinical value of lncRNAs as biomarkers for disease severity and prognostication also draws much attention. However, it should be admitted that this kind of preliminary findings need to be validated further. It will be desired to (1) develop a standardized lncRNAs testing system, including sample preparation, extraction, selection of appropriate endogenous controls (2); other statistical approaches such as predictive value, likelihood ratio, odd ratio and so on subjecting to the purpose of the biomarkers under investigation (3); conduct a multi-center study with a larger sample size to eliminate discrepancy such as ethnicity and sampling bias (4); perform a longitudinal study to validate lncRNAs as biomarkers for OA.

It appears that the modulation of the expression and activity of IncRNAs might be a novel strategy for OA management. Despite therapeutic nuclei acids hav been reported in OA treatment, several technical concerns including mechanism of action and an effective and specific delivery approach are not fully understood nor developed for OA application. Furthermore, the clinical application of lncRNAs-based therapy requires more stringent and robust investigation particularly safety issues including immunogenicity, cytotoxicity and long-term safety profile (207). In addition, the specificity of targeting lncRNAs is very important, and further studies are needed to avoid off-target side effects. Last but not least, a suitable target lncRNAs would lead to a more effective approach for OA treatment, and the focus of disease-specific lncRNAs described herein might draw some attention collaterally as the fields of gene-delivery and editing therapy develop.

## Author contributions

RL prepared the draft of the manuscript, which was revised by HS and WL. All authors have read and approved the final version of the manuscript.

## Funding

This work was partly supported by the General Research Fund (Ref. No. 24121622), Area of Excellence (Ref. No. AoE/M-402/20) and Research Matching Grant Scheme, University Grants Committee, Hong Kong; Start-up Fund, The Chinese University of Hong Kong, Hong Kong; Innovation and Technology Fund (Ref. No. PRP/090/20FX), Hong Kong; 2020 Rising Star Award provided by American Society for Bone and Mineral Research.

## Conflict of interest

The authors declare that the research was conducted in the absence of any commercial or financial relationships that could be construed as a potential conflict of interest.

## Publisher’s note

All claims expressed in this article are solely those of the authors and do not necessarily represent those of their affiliated organizations, or those of the publisher, the editors and the reviewers. Any product that may be evaluated in this article, or claim that may be made by its manufacturer, is not guaranteed or endorsed by the publisher.
